# Next-generation sequencing of *BRCA1* and *BRCA2* genes in Moroccan prostate cancer patients with positive family history

**DOI:** 10.1371/journal.pone.0254101

**Published:** 2021-07-09

**Authors:** Fatiha Salmi, Fatima Maachi, Amal Tazzite, Rachid Aboutaib, Jamal Fekkak, Houssine Azeddoug, Hassan Jouhadi

**Affiliations:** 1 Laboratory of Genetics and Molecular Pathology, Faculty of Medicine and Pharmacy, Hassan II University of Casablanca, Casablanca, Morocco; 2 Helicobacter Pylori and Gastric Pathologies Laboratory, Pasteur Institute of Morocco, Casablanca, Morocco; 3 Department of Urology, Ibn Rochd University Hospital Center, Casablanca, Morocco; 4 Molecular Biology Department, Anoual Laboratory, Casablanca, Morocco; 5 Faculty of Sciences-Biochemistry and Molecular Biology Laboratory, University Hassan II Casablanca, Casablanca, Morocco; 6 Mohammed VI Center for Cancer Treatment, Ibn Rochd University Hospital Center, Casablanca, Morocco; CNR, ITALY

## Abstract

Prostate cancer is the most common male cancer in Morocco. Although sporadic forms account for a large proportion of patients, familial forms of prostate cancer are observed in 20% of cases and about 5% are due to hereditary transmission. Indeed, germline mutations in *BRCA1*/*2* genes have been associated with prostate cancer risk. However, the spectrum of these mutations was not investigated in Moroccan Prostate cancer patients. Thereby, the aim of this study was to characterize and to estimate the prevalence of germline *BRCA1*/*2* mutations and large rearrangements in Moroccan patients with familial prostate cancer. The entire coding regions and intron/exon boundaries of *BRCA1* and *BRCA2* genes have been analyzed by next generation sequencing (NGS) in a total of 30 familial prostate cancer patients. Three pathogenic mutations were detected in four unrelated patients (13.3%). One *BRCA1* mutation (c.1953_1956delGAAA) and two *BRCA2* mutations (c.7234_7235insG and BRCA2ΔE12). In addition, sixty-three distinct polymorphisms and unclassified variants have been found. Early identification of germline *BRCA1*/*2* mutations may be relevant for the management of Moroccan prostate cancer patients.

## Introduction

Cancer is a serious public health problem in the world. About 18.1 million new cases and 9.6 million deaths have been reported by the International Agency for Research on Cancer (IARC) in 2018 [1]. Prostate cancer (PrCa) is one of the most commonly diagnosed cancers in men, especially in those aged over 50 [2]. Globally, there are 1,276,106 new cases and 358,989 deaths from PrCa per year [1]. In Morocco, about 3,990 new cases of PrCa have been diagnosed in 2018. Otherwise, it is the most common cancer of the genitourinary system and the most common cause of urological cancer death with 1,861 deaths [1].

Inherited mutations play a key role in the occurrence of PrCa. Epidemiological studies and segregation analysis have suggested a strong genetic origin of PrCa [3, 4]. The first linkage analysis in a series of prostate cancer patients have reported that 9% of familial prostate cancer cases are associated with alleles, located in a dominant susceptibility locus (HPC1), conferring high risk for prostate cancer with a penetrance of 88% at age 85 [5]. Moreover, differences in the incidence and outcome of PrCa observed among men of different race or ethnicity may confirm that some cases are partially attributed to genetic factors [6, 7]. Indeed, about 5 to 15% of PrCa cases are due to high-risk hereditary factors [8, 9]. Genome Wide Association Studies (GWAS) have revealed the association of a number of gene mutations with an increased PrCa risk such as *HOXB13*, *BRCA1*, *BRCA2*, *ATM*, *CHEK2*, *RAD51D*, *PALB2* and mismatch repair *(MMR)* genes [10, 11]. *BRCA1* and *BRCA2* are involved in maintaining of genome integrity [12]. *BRCA1* is a large gene located on chromosome 17q and composed of 22 exons which encode 1683 amino acids [13]. *BRCA2* gene has been located on chromosome 13q12-13 in 1995 and presents no homology with *BRCA1* gene. Currently, more than 2000 and 2400 distinct germline mutations have been described in *BRCA1* and *BRCA2* respectively [14]. Previous studies have observed that *BRCA1* and *BRCA2* pathogenic mutations carriers have 1.8 to 3.8-fold and 2.5 to 8.6-fold increased relative risk of developing PrCa by the age of ≤65 years old, respectively [15–19].

The relevance of *BRCA1*/2 mutations in patients with PrCa was not yet studied in Morocco. In fact, this work is the first Moroccan study investigating the spectrum of *BRCA1* and *BRCA2* germline mutations among Moroccan patients with a family history of PrCa using Next-generation sequencing (NGS) approach.

## Materials and methods

### Patients

This study involved 30 PrCa patients admitted to Mohammed VI center for cancer treatment in Casablanca, and selected according to the following criteria:

1. At least two cases of PrCa among first (father, brother) or second-degree relatives (grandfather, uncle).
2. Three cases of PrCa among first (father, son or brothers) or second-degree relatives (nephews, uncles on the maternal or paternal side).
3. Two cases of PrCa, diagnosed before age 55, in first-degree relatives (father, son or brothers) or second-degree relatives (nephews, uncles on the maternal or paternal side).

Each patient was asked to complete a questionnaire in order to obtain a complete family cancer history. Pathological features and medical data were collected from medical records. The study was performed in accordance with the Declaration of Helsinki protocols and was approved by the institutional ethical committee of BioMedical Research in Casablanca (CERBC) of the Faculty of Medicine and Pharmacy, Casablanca (Morocco), and written informed consent was obtained from each subject.

### Molecular analysis

#### DNA isolation

Genomic DNA was extracted from peripheral blood using a commercially available kit (Isolate II Genomic DNA Kit, Bioline). DNA concentration and purity were measured by NanoDrop 2000 Spectrophotometer and Qubit 3.0 Fluorometer (Thermo Fischer Scientific, Waltham, MA, USA).

#### Next generation sequencing

10 ng of DNA per sample was used to generate the sequencing library with the Ion PGM^™^ sequencing system and Oncomine^™^ BRCA Research Assay (Thermo Fisher Scientific, Waltham, MA, USA). This panel consists of two pools with 265 primer pairs covering complete coding sequence of *BRCA1* and *BRCA2* genes and splice site sequences at intron/exon junctions. PCR amplicons were partially digested by FuPa enzyme and then ligated to barcoded adapter. The generated amplicons were purified with AMPure^™^ XP Reagent (Beckman Coulter, Brea, CA, USA). After purification, libraries were quantified, diluted to 100 pM, and amplified through emulsion PCR on Ion OneTouch^™^ 2 System using Ion PGM^™^ Hi-Q^™^ View OT2 Kit (Thermo Fisher Scientific Waltham, MA, USA). Finally, NGS sequencing was performed on the Ion PGM^™^ sequencer using Ion PGM^™^ Hi-Q^™^ View Sequencing Kit (Thermo Fisher Scientific, Waltham, MA, USA).

#### Data analysis

Quality control of the sequencing data and their alignment to the HG19 human genome were conducted using the Ion Torrent Suite^™^ Software 5.0.5 (Thermo Fisher Scientific). The generated data were then analyzed by Torrent Variant Caller plugin version 5.0 (Thermo Fisher Scientific) in order to identify genetic variants and Ion Reporter^™^ software (Thermo Fisher Scientific) for variant annotation. The coverage depth was ≥ 250X.

All mutations were reported following the Human Genome Variation Society (HGVS) nomenclature (http://www.HGVS.org/varnomen) based on the coding sequences NM_007294.3 and NM_000059.3 for *BRCA1* and *BRCA2*, respectively. The variants were categorized as pathogenic or common polymorphisms or variant of uncertain significance (VUS) according to ClinVar database (https://www.ncbi.nlm.nih.gov/clinvar), Breast Cancer Information Core BIC (https://research.nhgri.nih.gov/bic/), the BRCA Exchange (https://brcaexchange.org), Universal Mutation Database (http://www.umd.be/BRCA1/, http://www.umd.be/BRCA2/), and Leiden Open (source) Variation Database (LOVD) (http://www.lovd.nl/3.0/home).

Unclassified variants were analyzed using *in silico* prediction tools: Polyphen (http://genetics.bwh.harvard.edu/pph2/) and Mutation taster (http://www.mutationtaster.org/).

Population frequency data are taken from various projects GnomAD (h ttps://gnomad.broadinstitute.org), TopMed (https://www.nhlbiwgs.org/topmed-whole-genome-sequencing-project-freeze-5b-phases-1-and-2) and ALFA (https://www.ncbi.nlm.nih.gov/snp/docs/gsr/alfa).

## Results

In this study, we have screened 30 familial prostate cancer patients for *BRCA1* and *BRCA2* mutations. The median age at PrCa diagnosis was 67.43 years (range 54–80). All patients present with high grade prostatic adenocarcinoma (grade IV), larger tumor size (≥ T2) and distant metastasis (see Table 1).

**Table 1:** Characteristics of studied Moroccan PrCa patients.

Code	Age D (years)	Ethnic Origin	Histology	Gleason Score	T	N	M	Stage	PSA (ng/ml)	Family History	*BRCA1/2* testing
Pr004	63	Arab	ADK	7	T4	Nx	M+	IV	7.5	a	-
Pr419	61	Arab	ADK	9	T4	N+	M+	IV	1000.00	a	*BRCA1+*
Pr423	65	Arab	ADK	6	T3	Nx	M+	III	10.23	b	-
Pr425	69	Amazigh	ADK	7	T4	Nx	M+	IV	300.00	a	-
Pr438	74	Arab	ADK	5	T3	Nx	M+	III	58.00	a	-
Pr502	62	Arab	ADK	6	T4	Nx	M+	IV	154.00	a	-
Pr509	70	Amazigh	ADK	6	T3	Nx	M0	III	41.00	a	-
Pr512	64	Arab	ADK	7	T4	Nx	Mx	IV	216.00	b	-
Pr528	54	Amazigh	ADK	4	T2	Nx	Mx	II	2.80	a	-
Pr529	73	Arab	ADK	8	T3	Nx	Mx	III	386.00	a	-
Pr531	74	Arab	ADK	7	T2	N0	M0	II	16.00	a	-
Pr533	69	Arab	ADK	7	T3	N0	M0	III	05.20	b	-
Pr552	70	Arab	ADK	7	T4	Nx	M+	IV	31.67	a	*BRCA2+*
Pr559	74	Amazigh	ADK	7	T4	Nx	M+	IV	106.00	a	-
Pr561	73	Amazigh	ADK	9	T4	Nx	M+	IV	1710.00	a	-
Pr562	70	Arab	ADK	7	T3	Nx	M0	III	10.83	a	-
Pr569	72	Arab	ADK	8	T4	Nx	M+	IV	2000.00	a	*BRCA2+*
Pr570	75	Arab	ADK	7	T3	Nx	M+	IV	13.21	a	-
Pr579	71	Amazigh	ADK	8	T4	Nx	M+	IV	420.00	a	-
Pr605	65	Arab	ADK	7	T4	Nx	M+	IV	09.63	a	*BRCA2+*
Pr610	72	Amazigh	ADK	6	T3	Nx	M0	III	41.00	a	-
Pr612	55	Arab	ADK	7	T3	Nx	Mx	III	18.74	a	-
Pr613	67	Arab	ADK	6	T4	Nx	M+	IV	106.00	a	-
Pr617	58	Arab	ADK	8	T4	Nx	M+	IV	60.00	a	-
Pr713	74	Amazigh	ADK	7	T4	Nx	M+	IV	106.24	a	-
Pr722	80	Arab	ADK	8	T4	Nx	M+	IV	238.00	a	-
Pr725	74	Amazigh	ADK	7	T4	N3	M+	IV	45.00	a	-
Pr731	60	Arab	ADK	7	T4	N3	M+	IV	5.43	a	-
Pr733	74	Arab	ADK	7	T4	Nx	M+	IV	36.00	a	-
Pr803	57	Arab	ADK	7	T4	N1	M+	IV	6.00	a	-

Age D: Age at diagnostic, ADK: Adenocarcinoma, T: Size of the tumor, N: Node involvement, M: Metastasis, PSA: Protein Specific Antigen, a: At least two cases of PrCa among first (father, brother) or second-degree relatives (grandfather, uncle), b: Three cases of PrCa among first (father, son or brothers) or second-degree relatives (nephews, uncles on the maternal or paternal side).

**Fig 1:**
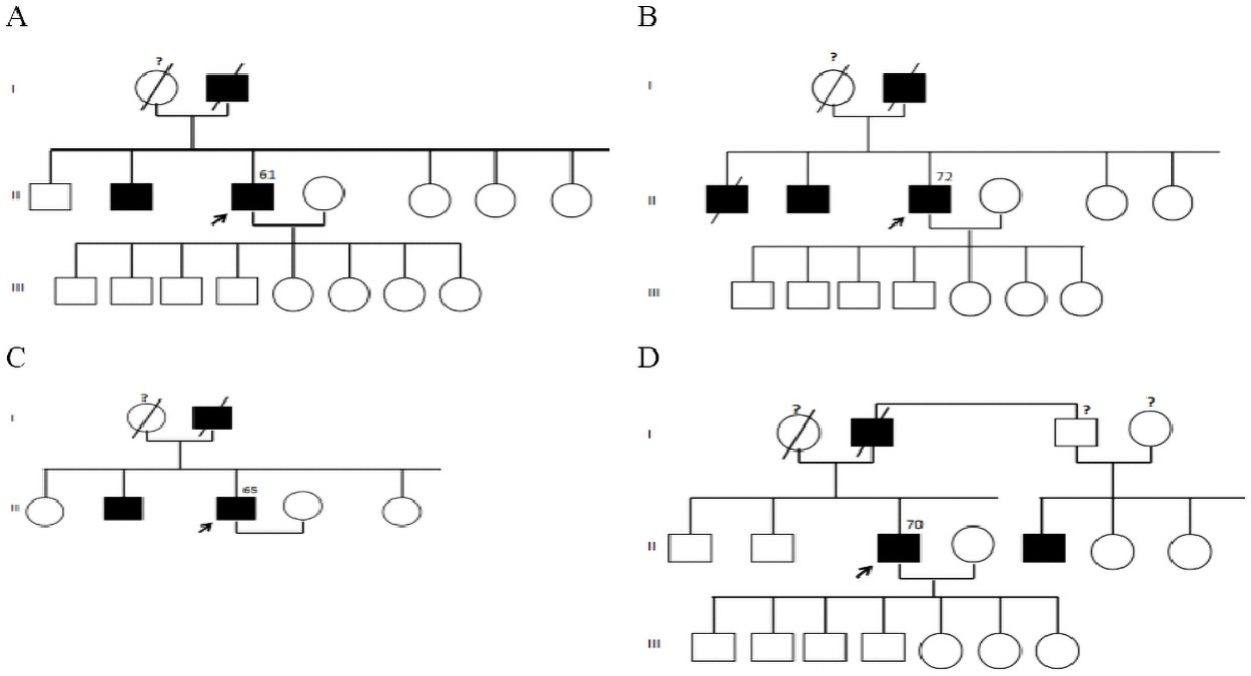
Pedigrees of patients carrying *BRCA1*/2 deleterious mutations. (A) *BRCA1* c.1953_1956delGAAA. (B) *BRCA2* c.7234_7235insG. (C and D) *BRCA2* delExon12. The numbers indicate age of patients at diagnosis.

Three *BRCA1* and *BRCA2* pathogenic mutations have been detected in four unrelated patients (see Table 2). One patient was found to carry a mutation in *BRCA1* gene and three in *BRCA2* gene. Therefore, the combined mutation frequency was 13.3% (4/30). The first mutation was identified in exon 10 of *BRCA1* gene. A four nucleotide deletion called c.1953_1956delGAAA at the cDNA level and p.Lys653SerfsX47 (K653SfsX47) at the protein level. The second mutation was a small insertion detected in exon 14 of *BRCA2* gene (c.7234_7235insG (7463insG)) inducing a shift in the reading frame. Finally, a whole-exon 12 deletion (BRCA2ΔE12) have been identified in *BRCA2* gene. All three pathogenic mutations were found in patients with a strong family history of PrCa. The pedigree analysis revealed at least two affected family members with PrCa over two or three generations (Fig 1).

**Table 2:** Pathogenic *BRCA1/2* mutations identified in the present study.

Gene	Exon	Nombre of patients	Genotype	Amino acid change	Mutation Type
*BRCA1*	10	1	c.1953_1956delGAAA	p.Lys653SerfsTer47	Frameshift Deletion
*BRCA2*	14	1	c.7234_7235insG	p.Thr2412SerfsTer2	Frameshift Insertion
*BRCA2*	12	2	-	-	Exon Deletion

Additionally, sixty-three non-pathogenic *BRCA1* and *BRCA2* mutations were detected in this study (Table 3). Twenty eight mutations (14 missense mutations, 4 synonymous substitutions, 9 intronic variants and one non-framshift deletion) and thirty-five mutations (15 missense mutations, 11 synonymous substitutions and 9 intronic variants) were identified in *BRCA1* and *BRCA2* genes respectively.

**Table 3:** *BRCA1*/2 polymorphisms and unclassified variants.

Gene	Nucleotide	Protein change	Mutation Type	Number of patients	Allele frequency	Clinical significance (Clinvar)	Co-occurrence with a pathogenic mutation
***BRCA1***	c.301+55G>A	p.?	Intron Variant	01	0.00006^a^	Benign	No
	c.442-34C>T	p.?	Intron Variant	06	0.17302 ^b^	Benign	Yes
	c.536A>G	p.Tyr179Cys	Missense	02	0.00026 ^b^	Benign	No
	c.548-58delT	p.?	Intron Variant	06	0.28382 ^a^	Benign	Yes
	c.1456T>C	p.Phe486Leu	Missense	02	0.00041 ^a^	Benign	No
	c.1648A>C	p.Asn550His	Missense	02	0.00026 ^b^	Benign	No
	c.1846_1848delTCT	p.Ser616del	Nonframshift deletion	01	0.00027 ^b^	Benign	No
	c.2077G>A	p.Asp693Asn	Missense	02	0.05843 ^b^	Benign	No
	c.2082C>T	p.Ser694 =	Synonymous	11	0.35262 ^b^	Benign	Yes
	c.2311T>C	p.Leu771 =	Synonymous	11	0.34739 ^b^	Benign	Yes
	c.2521C>T	p.Arg841Trp	Missense	03	0.00167 ^b^	Begnin	No
	c.2612C>T	p.Pro871Leu	Missense	18	0.35648 ^c^	Begnin	Yes
	c.3113A>G	p.Glu1038Gly	Missense	11	0.34827 ^b^	Begnin	Yes
	c.3119G>A	p.Ser1040Asn	Missense	04	0.01315 ^b^	Begnin	Yes
	c.3548A>G	p.Lys1183Arg	Missense	11	0.35268 ^b^	Begnin	Yes
	c.4308T>C	p.Ser1436Ser	Synonymous	11	0.34852 ^b^	Begnin	Yes
	c.4358-2885G>A	p.?	Intron Variant	11	0.30184 ^a^	Begnin	Yes
	c.4600G>A	p.Val1534Met	Missense	01	0.00039 ^b^	Begnin	No
	c.4837A>G	p.Ser1613Gly	Missense	10	0.33675 ^c^	Begnin	Yes
	c.4882A>G	p.Met1628Val	Missense	01	0.00002 ^b^	Conflicting interpretations of pathogenicity	No
	c.4900A>G	p.Ser1634Gly	Missense	01	-	Uncertain significance	No
	c.4987-92A>G	p.?	Intron Variant	11	0.30294 ^a^	Begnin	Yes
	c.4987-68A>G	p.?	Intron Variant	11	0.30295 ^a^	Begnin	Yes
	c.5117G>C	p.Gly1706Ala	Missense	03	0.00005 ^b^	Begnin	No
	c.5152+20T>A	p.?	Intron Variant	01	0.00016 ^b^	Benign/Likely benign	No
	c.5152+85delT	p.?	Intron Variant	03	0.02646 ^a^	Begnin	Yes
	c.5175A>G	p.Glu1725 =	Synonymous	01	0.000096 ^b^	Likely benign	No
	c.5215+66G>A	p.?	Intron Variant	11	0.29599 ^a^	Begnin	Yes
***BRCA2***	c.-26G>A	p.?	Intron Variant	06	0.24553 ^b^	Benign	Yes
	c.231T>G	p.Thr77 =	Synonymous	01	0.00047 ^b^	Benign	No
	c.425+67A>C	p.?	Intron Variant	02	0.03973 ^a^	Benign	No
	c.681+56C>T	p.?	Intron Variant	10	0.20076 ^a^	Benign	Yes
	c.1910-74T>C	p.?	Intron Variant	01	0.18998 ^a^	Benign	No
	c.865A>C	p.Asn289His	Missense	02	0.03968 ^a^	Benign	No
	c.1114A>C	p.Asn372His	Missense	13	0.27964 ^b^	Benign	Yes
	c.1365A>G	p.Ser455 =	Synonymous	02	0.05231 ^b^	Benign	No
	c.1627C>A	p.His543Asn	Missense	01	0.0 ^c^	Uncertain significance	No
	c.1788T>C	p.Asp596 =	Synonymous	01	0.00203 ^b^	Benign	No
	c.2229T>C	p.His743 =	Synonymous	02	0.05272 ^b^	Benign	No
	c.2786T>C	p.Leu929Ser	Missense	01	0.00073 ^b^	Benign	No
	c.2960A>T	p.Asn987Ile	Missense	01	0.00073 ^b^	Benign	No
	c.2971A>G	p.Asn991Asp	Missense	02	0.05409 ^b^	Benign	No
	c.3396A>G	p.Lys1132 =	Synonymous	08	0.29462 ^b^	Benign	Yes
	c.3516G>A	p.Ser1172 =	Synonymous	01	0.00168 ^a^	Benign	Yes
	c.3807T>C	p.Val1269 =	Synonymous	15	0.17460 ^b^	Benign	Yes
	c.4563A>G	p.Leu1521 =	Synonymous	30	0.02311 ^a^	Benign	Yes
	c.4585G>A	p.Gly1529Arg	Missense	01	0.00039 ^b^	Benign	No
	c.5312G>A	p.Gly1771Asp	Missense	01	0.00022 ^a^	Benign	No
	c.6513G>C	p.Val2171 =	Synonymous	30	0.02313 ^a^	Benign	Yes
	c.7242A>G	p.Ser2414 =	Synonymous	08	0.22464 ^a^	Benign	Yes
	c.7397T>C	p.Val2466Ala	Missense	30	0.00512 ^b^	Benign	Yes
	c.7435+53C>T	p.?	Intron Variant	02	0.03924 ^a^	Benign	No
	c.7806-14T>C	p.?	Intron Variant	19	0.47713 ^b^	Benign	Yes
	c.7806-40A>G	p.?	Intron Variant	01	0.00847 ^a^	Benign	No
	c.7954G>A	p.Val2652Met	Missense	01	0.000004 ^b^	Uncertain significance	No
	c.8331+109G>A	p.?	Intron Variant	01	0.00779 ^a^	Benign	No
	c.8460A>C	p.Val2820 =	Synonymous	01	0.00311 ^b^	Benign	No
	c.8503T>C	p.Ser2835Pro	Missense	02	0.00055 ^b^	Benign	No
	c.8687G>A	p.Arg2896His	Missense	01	0.00002 ^b^	Conflicting interpretations of pathogenicity	No
	c.8755-66T>C	p.?	Intron Variant	18	0.48723 ^c^	Benign	Yes
	c.8830A>T	p.Ile2944Phe	Missense	01	0.00295 ^b^	Benign	No
	c.9364G>A	p.Ala3122Thr	Missense	01	0.00010 ^b^	Conflicting interpretations of pathogenicity	Yes
	c.10234A>G	p.Ile3412Val	Missense	05	0.02331 ^b^	Benign	No

^a^: TOPMED;

^b^: GnomAD_exome;

^c^: ALFA Project

Regarding previously reported VUS, in silico analysis was performed for six detected missense mutations reported as VUS or with conflicting interpretations of pathogenecity in Clinvar database (see Table 4). Two mutations c.7954G>A and c.9364G>A in *BRCA2* gene were predicted to be implicated in the disease.

**Table 4:** Protein prediction of uncertain significance *BRCA1/BRCA2* variants.

Gene	Nucleotide	Amino Acid change	Mutation taster	Polyphen2
*BRCA1*	c.4882A>G	M1628V	Polymorphism	BENIGN with a score of 0.001
	c.4900A>G	S1634G	Polymorphism	BENIGN with a score of 0.081
*BRCA2*	c.1627C>A	H543N	Polymorphism	BENIGN with a score of 0.003
	c.7954G>A	V2652M	Disease causing	PROBABLY DAMAGING with a score of 1.000
	c.8687G>A	R2896H	Polymorphism	BENIGN with a score of 0.004
	c.9364G>A	A3122T	Disease causing	PROBABLY DAMAGING with a score of 1.000

## Discussion

Germline mutations in *BRCA1* and *BRCA2* genes have been associated with a high risk of ovarian/breast and prostate cancers. Indeed, 13–18% of hereditary ovarian cancer cases [20–22], and around 5% of hereditary breast cancer cases are due to *BRCA1* and *BRCA2* mutations [23]. As well, the frequencies of both mutations in PrCa patients are 0.9% and 2.2% respectively [24]. In Morocco, *BRCA1* and *BRCA2* mutations have been extensively investigated for breast cancer but not so for prostate cancer. Thereby, our study is the first to describe inherited *BRCA1* and *BRCA2* mutation spectrum and prevalence in Moroccan PrCa patients using the high-throughput sequencing technique.

According to our findings, the frequency of deleterious *BRCA1* (3.33%) and *BRCA2* (10%) mutations was 13.33%. This frequency appears to be high compared to those observed in other populations such as in the UK (4% to 6%) [6, 25, 26], Ashkenazi Jews (1.4% to 5.2%) [27–35], Finns (3.3%) [36], in Israel (3.8%) [37], and Portuguese (5.2%) [38]. Considering only *BRCA2* mutations, some studies have reported frequencies ranging from 1% to 7% [18, 39–45]. While for *BRCA1* mutations, frequencies of 0.1%, 0.4% and 0.45% have been reported in Spain, Poland and UK respectively [16, 46–48]. The variations in *BRCA1*/2 mutation frequencies across populations may be due to variation in study sample size and inclusion criteria or patients’ ethnic background.

Diversity of mutations and their distribution throughout both genes complicate the initial mutation screening. Overall, each family has its "private" mutation. However, some recurrent mutations have been identified in Ashkenazi Jews (*BRCA1* 185delAG, *BRCA1* 5382insC and *BRCA2* 6174delT) [41–43]. Other founder mutations have also been reported in Portugal [46], Germany [44], Poland [48], Canada [45], Turkey [43], Iceland [42], and USA [40]. In the present study, we detected three pathogenic mutations in four patients from different regions of Morocco. The first mutation is a frameshift deletion in exon 10 of *BRCA1* gene (c.1953_1956delGAAA). The second mutation is a frameshift insertion that is located in exon 14 of *BRCA2* gene (c.7234_7235insG) and the last one is a deletion of entire exon 12 of *BRCA2* gene. All these pathogenic variants and their impact have been previously reported in BIC and ClinVar databases.

First, the c.1953_1956delGAAA (p.Lys653SerfsTer47) mutation, also known as 2072_2075delGAAA or 2072delGAAA or 2072del4 under other nomenclatures, is a deletion of four nucleotide bases in exon 10 which is reported for the first time in the Moroccan population but previously identified in breast and/or ovarian cancer patients from other populations [20, 49–59]. This deletion changes the reading frame and creates a premature stop codon at position 47.

The second pathogenic variant (c.7234_7235insG) also found in *BRCA2* gene is an insertion of guanine between nucleotides 7234 and 7235 in exon 14 which causes a shift in reading frame (Stop2413) and, as a consequence, the production of premature truncated protein. This mutation has been previously found in several breast/ovarian cancer families. It was first reported by Esteban Cardeñosa et al. [53] in a single Eastern Spanish family with breast/ovarian cancers. Next, two unrelated Moroccan patients with familial breast cancer were found to carry it in the study by Tazzite et al. [60]. Later, this mutation was also identified by De Juan Jiménez et al. [55] in a single patient with familial breast and ovarian cancer, and by De Juan et al. [61] in a man with breast cancer who did not have family history. Recently, it was described for a second time in a Moroccan breast cancer family [62]. It is important to note that this mutation is described for the first time in a familial PrCa case.

The last mutation was a complete deletion of *BRCA2* exon 12 that was found in two unrelated PrCa patients. This isoform is known as BRCA2ΔE12 or BRCA2 del 12. Characterization of this genomic breakpoint or large genomic rearrangement (LGR) revealed a deletion of 96bp, which is similar to the deletion previously described [63, 64]. This mutation is an in-frame deletion that should result in the production of a 32 amino acid shortened protein. Many *BRCA1* and *BRCA2* LGRs have been associated with hereditary breast, ovarian and prostate cancers [65–68]. Their frequencies vary across different population. In general, they account for 4 to 28% of all *BRCA1*/*2* mutations [69]. The majority of these variants occur within *BRCA1*, probably because of the high rate of Alu elements in this gene [70, 71]. To our knowledge, no specific function has been attributed to exon 12 of *BRCA2* gene. However, its deletion may affect the functions of the adjacent domains or change the structure of the entire polypeptide. Speculatively, BRCA2Δ12 mutation may alter the ability of *BRCA2* protein to repair DNA because it is located downstream of exon 11 which contains domains essential for interaction with *RAD51* [63]. The detection of this mutation in two unrelated patients supported by the observation of common shared polymorphisms and unclassified variants namely c.3807T>C, c.6513G>C, c.4563A>G and c.7397T>C suggest that BRCA2Δ12 mutation may be a recurrent mutation in our population. Larger studies are needed to confirm this finding.

Several studies have been interested in familial PrCa specifically for *BRCA1* and *BRCA2* gene testing. Wilkens et al. [27] have tested three *BRCA* founder mutations in Ashkenazi Jewish families with PrCa. Their results showed that only one unaffected participant carried *BRCA2* 6174delT mutation [27]. Similarly, 38 PrCa families from UK have not been found to have pathogenic *BRCA1* mutations but two *BRCA2* germline mutations (6710delACAA and 5531delTT) were found in young patients [25]. In addition, Sinclair et al. [40] have screened 43 individuals with positive family history of PrCa and found one *BRCA2* missense mutation (C1206A). In a large German cohort, mutation screening revealed five *BRCA2* mutations (c.1813_14_insA, c.3847delGT, c.4449delA, c.6037A>T, c.7495C>T) [44].

Interestingly, all cases in the present cohort show higher grade prostatic adenocarcinoma with higher T stage and high Gleason score. These results are consistent with previous studies showing that male PrCa patients tend to present with more aggressive tumors [72, 73]. The histopathological analysis of 20 tumor tissue samples from patients with hereditary PrCa revealed that *BRCA* mutation carriers have higher frequency of grade tumors with a Gleason score ≥8 than non carriers (P = 0.012) [74]. This finding was consistent with those of a large study by Castro et al. [26] who found that PrCa patients with *BRCA* mutations were more likely to have T3-T4 tumors (P = 0.003), Gleason score of at least 8 (P = 0.00003), lymph node invasion (P = 0.00005) and distant metastases at the initial diagnosis (P = 0.005) compared to *BRCA*-negative patients. Recently, Petrovics et al. [75] have confirmed these observations. They observed that frequencies of deleterious *BRCA* mutation, particularly *BRCA2* mutations, are higher in patients with advanced PrCa [75].

As well as deleterious mutations, *BRCA1* and *BRCA2* variants of unknown/uncertain significance (VUS) significantly arouse interest amongst the geneticists. Their clinical implication is still unclear which complicates genetic counseling. Complete gene and genome sequencing by NGS increases the number of discovered VUS. In the present work, we have identified 63 variants including neutral polymorphisms and VUS that have been previously reported. Twenty-nine of these are missense mutations (14 in *BRCA1* and 15 in *BRCA2* genes). Thirty-two variants have already been reported as polymorphisms. In fact, previous studies have identified some single-nucleotide polymorphisms associated with prostate cancer risk including c.442-34C>T (rs799923), c.1067A>G (rs1799950), c.4837A>T (rs1799966), c.4357+117G>C (rs3737559) for *BRCA1* gene and c.1114A>C (rs144848) for *BRCA2* gene [76, 77].

This is the first report of the prevalence of *BRCA1* and *BRCA2* mutations in Moroccan PrCa patients who had a family history. To conclude, although our results are considered preliminary due to the small sample size, they underline the importance of *BRCA1* and *BRCA2* genetic screening in hereditary PrCa. Hence, early identification of germline mutations in *BRCA1* and *BRCA*2 genes may be relevant for management of patients with PrCa and also for preventing future cancers in their relatives.

## Supporting information

S1 Table BRCA1/2 polymorphisms and unclassified variants. (PDF)
