# Supplementary material for: Next-generation sequencing of BRCA1 and BRCA2 genes in Moroccan prostate cancer patients with positive family history
Source: PLoS One. 2021 Jul 9;16(7):e0254101. doi: 10.1371/journal.pone.0254101 (PMC8270444; doi:10.1371/journal.pone.0254101)
Supplement: S1 Table — (PDF) [file pone.0254101.s001.pdf]

S1 Table: *BRCA1*/2 polymorphisms and unclassified variants

Gene	Nucleotide	Protein change	Amino Acid change	Mutation Type	Number of patients	Allele frequency	Clinical significance (Clinvar)	Global minor allele frequency (GMAF)	Co-occurrence with a pathogenic mutation
**BRCA1**	c.301+55G>A	p.?	Non-coding	Intron Variant	01	0.00006 (TOPMED)	Benign	-	No
	c.442-34C>T	p.?	Non-coding	Intron Variant	06	0.17302 (GnomAD_exome)	Benign	0.09864 (A)	Yes
	c.536A>G	p.Tyr179Cys	Y179C	Missense	02	0.00026 (GnomAD_exome)	Benign	-	No
	c.548-58delT	p.?	Non-coding	Intron Variant	06	0.283822 (TOPMED)	Benign	0.33486 (AAAAAA)	Yes
	c.1456T>C	p.Phe486Leu	F486L	Missense	02	0.000406 (TOPMED)	Benign	0.00020 (G)	No
	c.1648A>C	p.Asn550His	N550H	Missense	02	0.000259 (GnomAD_exome)	Benign	0.00020 (G)	No
	c.1846_1848del TCT	p.Ser616del	S616del	Nonframshift deletion	01	0.000267 (GnomAD_exome)	Benign	0.00120 (AGA)	No
	c.2077G>A	p.Asp693Asn	D693N	Missense	02	0.058433 (GnomAD_exome)	Benign	0.03355 (T)	No
	c.2082C>T	p.Ser694=	S694S	Synonymous	11	0.352622 (GnomAD_exome)	Benign	0.33646 (A)	Yes
	c.2311T>C	p.Leu771=	L771L	Synonymous	11	0.347393 (GnomAD_exome)	Benign	0.33526 (G)	Yes

	c.2521C>T	p.Arg841Trp	R841W	Missense	03	0.001673 (GnomAD_exome)	Begnin	0.00220 (A)	No
	c.2612C>T	p.Pro871Leu	P871L	Missense	18	0.356483 (ALFA Project)	Begnin	0.45607 (G)	Yes
	c.3113A>G	p.Glu1038Gly	E1038G	Missense	11	0.348270 (GnomAD_exome)	Begnin	0.33566 (C)	Yes
	c.3119G>A	p.Ser1040Asn	S1040N	Missense	04	0.013154 (GnomAD_exome)	Begnin	0.00978 (T)	Yes
	c.3548A>G	p.Lys1183Arg	K1183R	Missense	11	0.352682 (GnomAD_exome)	Begnin	0.35264 (C)	Yes
	c.4308T>C	p.Ser1436Ser	S1436S	Synonymous	11	0.348519 (GnomAD_exome)	Begnin	0.33626 (G)	Yes
	c.4358-2885G>A	p.?	Non-coding	Intron Variant	11	0.301836 (TOPMED)	Begnin	0.35344 (T)	Yes
	c.4600G>A	p.Val1534Met	V1534M	Missense	01	0.000386 (GnomAD_exome)	Begnin	0.00160 (T)	No
	c.4837A>G	p.Ser1613Gly	S1613G	Missense	10	0.336748 (ALFA Project)	Begnin	0.35583 (C)	Yes
	c.4882A>G	p.Met1628Val	M1628V	Missense	01	0.000020 (GnomAD_exome)	Conflicting interpretations of pathogenicity	-	No
	c.4900A>G	p.Ser1634Gly	S1634G	Missense	01	-	Uncertain significance	-	No

	c.4987-92A>G	p.?	Non-coding	Intron Variant	11	0.302935 (TOPMED)	Begnin	0.35463 (C)	Yes
	c.4987-68A>G	p.?	Non-coding	Intron Variant	11	0.302951 (TOPMED)	Begnin	0.35463 (C)	Yes
	c.5117G>C	p.Gly1706Ala	G1706A	Missense	03	0.000048 (GnomAD_exome)	Begnin	0.00020 (G)	No
	c.5152+20T>A	p.?	Non-coding	Intron Variant	01	0.000159 (GnomAD_exome)	Benign/Likely benign	0.00020 (T)	No
	c.5152+85delT	p.?	Non-coding	Intron Variant	03	0.026456 (TOPMED)	Begnin	0.02516 (AAAA)	Yes
	c.5175A>G	p.Glu1725=	E1725E	Synonymous	01	0.000096 (GnomAD_exome)	Likely benign	-	No
	c.5215+66G>A	p.?	Non-coding	Intron Variant	11	0.295991 (TOPMED)	Begnin	0.34245 (T)	Yes
**BRCA2**	c.-26G>A	p.?	Non-coding	Intron Variant	06	0.245533 (GnomAD_exome)	Benign	0.20927 (A)	Yes
	c.231T>G	p.Thr77=	T77T	Synonymous	01	0.000465 (GnomAD_exome)	Benign	0.00300 (G)	No
	c.425+67A>C	p.?	Non-coding	Intron Variant	02	0.039731 (TOPMED	Benign	0.07428 (C)	No
	c.681+56C>T	p.?	Non-coding	Intron Variant	10	0.200760 (TOPMED)	Benign	0.18590 (T)	Yes
	c.1910-74T>C	p.?	Non-coding	Intron Variant	01	0.189977 (TOPMED)	Benign	0.17452 (C)	No
	c.865A>C	p.Asn289His	N289H	Missense	02	0.039684 (TOPMED)	Benign	0.07368 (C)	No
	c.1114A>C	p.Asn372His	N372H	Missense	13	0.279642 (GnomAD_exome)	Benign	0.24940 (C)	Yes

	c.1365A>G	p.Ser455=	S455S	Synonymous	02	0.052310 (GnomAD_exome)	Benign	0.07368 (G)	No
	c.1627C>A	p.His543Asn	H543N	Missense	01	0.0 (ALFA Project)	Uncertain significance	-	No
	c.1788T>C	p.Asp596=	D596D	Synonymous	01	0.002032 (GnomAD_exome)	Benign	0.00819 (C)	No
	c.2229T>C	p.His743=	H743H	Synonymous	02	0.052715 (GnomAD_exome)	Benign	0.07348 (C)	No
	c.2786T>C	p.Leu929Ser	L929S	Missense	01	0.000733 (GnomAD_exome)	Benign	0.00200 (C)	No
	c.2960A>T	p.Asn987Ile	N987I	Missense	01	0.000729 (GnomAD_exome)	Benign	0.00200 (T)	No
	c.2971A>G	p.Asn991Asp	N991D	Missense	02	0.054087 (GnomAD_exome)	Benign	0.08007 (G)	No
	c.3396A>G	p.Lys1132=	K1132K	Synonymous	08	0.294623 (GnomAD_exome)	Benign	0.26677 (G)	Yes
	c.3516G>A	p.Ser1172=	S1172	Synonymous	01	0.001680 (TOPMED)	Benign	0.00160 (A)	Yes
	c.3807T>C	p.Val1269=	V1269V	Synonymous	15	0.174603 (GnomAD_exome)	Benign	0.16813 (C)	Yes
	c.4563A>G	p.Leu1521=	L1521L	Synonymous	30	0.023111 (TOPMED)	Benign	0.02596 (A)	Yes
	c.4585G>A	p.Gly1529Arg	G1529R	Missense	01	0.000391	Benign	0.00060 (A)	No

						(GnomAD_exome)			
	c.5312G>A	p.Gly1771Asp	G1771D	Missense	01	0.000223 (TOPMED)	Benign	0.00020 (A)	No
	c.6513G>C	p.Val2171=	V2171V	Synonymous	30	0.023127 TOPMED)	Benign	0.02596 (G)	Yes
	c.7242A>G	p.Ser2414=	S2414S	Synonymous	08	0.224635 (TOPMED)	Benign	0.23263 (G)	Yes
	c.7397T>C	p.Val2466Ala	V2466A	Missense	30	0.005124 (GnomAD_exome)	Benign	0.02416 (T)	Yes
	c.7435+53C>T	p.?	Non-coding	Intron Variant	02	0.039238 (TOPMED)	Benign	0.07248 (T)	No
	c.7806-14T>C	p.?	Non-coding	Intron Variant	19	0.477129 (GnomAD_exome)	Benign	0.46845 (T)	Yes
	c.7806-40A>G	p.?	Non-coding	Intron Variant	01	0.008473 (TOPMED)	Benign	0.00938 (G)	No
	c.7954G>A	p.Val2652Met	V2652M	Missense	01	0.000004 (GnomAD_exome)	Uncertain significance	-	No
	c.8331+109G>A	p.?	Non-coding	Intron Variant	01	0.007789 (TOPMED)	Benign	0.00819 (A)	No
	c.8460A>C	p.Val2820=	V2820V	Synonymous	01	0.003107 (GnomAD_exome)	Benign	0.01438 (C)	No
	c.8503T>C	p.Ser2835Pro	S2835P	Missense	02	0.000549 (GnomAD_exome)	Benign	0.00140 (C)	No
	c.8687G>A	p.Arg2896His	R2896H	Missense	01	0.000016 (GnomAD_exome)	Conflicting interpretations of pathogenicity	-	No

	c.8755-66T>C	p.?	Non-coding	Intron Variant	18	0.487228 (ALFA Project)	Benign	0.48842 (T)	Yes
	c.8830A>T	p.Ile2944Phe	I2944F	Missense	01	0.002950 (GnomAD_exome)	Benign	0.00899 (T)	No
	c.9364G>A	p.Ala3122Thr	A3122T	Missense	01	0.000103 (GnomAD_exome)	Conflicting interpretations of pathogenicity	-	Yes
	c.10234A>G	p.Ile3412Val	I3412V	Missense	05	0.023306 (GnomAD_exome)	Benign	0.04493 (G)	No
